# Non-classical disproportionation revealed by photo-chemically induced dynamic nuclear polarization NMR

**DOI:** 10.5194/mr-2-281-2021

**Published:** 2021-05-07

**Authors:** Jakob Wörner, Jing Chen, Adelbert Bacher, Stefan Weber

**Affiliations:** 1 Institute of Physical Chemistry, Albert-Ludwigs-Universität Freiburg, Freiburg, 79104, Germany; 2 Department of Chemistry, Technical University of Munich, Garching, 85748, Germany

## Abstract

Photo-chemically induced dynamic nuclear polarization (photo-CIDNP) was used to observe the light-induced disproportionation reaction of 6,7,8-trimethyllumazine starting out from its triplet state to generate a pair of radicals comprising a one-electron reduced and a one-electron oxidized species. Our evidence is based on the measurement of two marker proton hyperfine couplings, $A_{\mathrm{iso}}$ (H(6 α )) and $A_{\mathrm{iso}}$ (H(8 α )), which we correlated to predictions from density functional theory. The ratio of these two hyperfine couplings is reversed in the oxidized and the reduced radical species. Observation of the dismutation reaction is facilitated by the exceptional C–H acidity of the methyl group at position 7 of 6,7,8-trimethyllumazine and the slow proton exchange associated with it, which leads to NMR-distinguishable anionic (TML ${}^{-}$ ) and neutral (TMLH) protonation forms.

## 1 Introduction

**Scheme 1:**
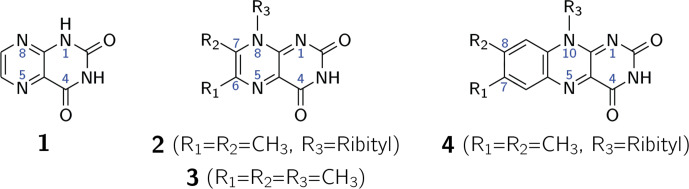
Lumazine (1), 6,7-dimethyl-8-ribityllumazine (2), 6,7,8-trimethyllumazine (3) and riboflavin (4).

The first intentional synthesis of pteridine-2,4(1H,3H)-dione (1; see Scheme 1) was described by Kühling in 1894 (Kühling, 1894, 1895). Due to its blueish-green fluorescence even in very dilute aqueous solutions, the compound was named lumazine (Kuhn and Cook, 1937). More than 70 years after its first synthesis, 1 was recognized as a natural product and isolated from male ants of the species *Formica polyctena* (Schmidt and Viscontini, 1967) and more recently also from the leaves of *Brassica juncea* L. (brown mustard) (Sharma et al., 2018). Unsubstituted lumazine (1) is not widely distributed in nature (Daniels et al., 2019); related compounds substituted at positions 6, 7 and 8 are of greater biological significance. Importantly, 6,7-dimethyl-8-ribityllumazine (2) is the direct biosynthetic precursor of riboflavin (4; vitamin B ${}_{2}$ ) (Masuda, 1957; Maley and Plaut, 1959; Plaut, 1960, 1963) whose derivatives flavin mononucleotide (FMN) and flavin adenine dinucleotide (FAD) are universally distributed and are involved in an amazing variety of essential biological processes; for recent reviews, see Walsh and Wencewicz (2013) and Piano et al. (2017). The biosynthetic precursor 2 affords riboflavin by a mechanistically unique dismutation that is catalyzed by the enzyme riboflavin synthase and does not require any cosubstrates or cofactors (Plaut and Harvey, 1971). Even more surprisingly, the dismutation affording 4 from 2 can also proceed non-enzymatically in neutral or acidic aqueous solution under an inert gas atmosphere (Rowan and Wood, 1963; Kis et al., 2001).

6,7-Dimethyl-8-ribityllumazine (2) acts as cofactor of lumazine protein (LumP), an optical transponder involved in bioluminescence of certain marine bacteria (Koka and Lee, 1979; Small et al., 1980). More recently, 2 has also been found in two members of the photolyase/cryptochrome protein family, namely cryptochrome B (CryB) from *Rhodobacter* (*R*.) *sphaeroides* (Geisselbrecht et al., 2012) and the photolyase-related protein B (PhrB) from *Agrobacterium* (*A*.) *tumefaciens* (Zhang et al., 2013). Both proteins belong to the new subclass of FeS bacterial cryptochromes and photolyases (BCP), also called CryPro. *R. sphaeroides* CryB controls light-dependent and singlet-oxygen-dependent gene expression of the photosynthetic apparatus (Frühwirth et al., 2012). Similar to *A. tumefaciens* PhrB, CryB has also repair activity for (6–4) photoproducts in photodamaged DNA (von Zadow et al., 2016). It has been speculated that 2 acts as antenna chromophore in this protein class (Geisselbrecht et al., 2012). This additional cofactor absorbs at shorter wavelengths ( $\lambda_{max}$ = 420 nm) than the essential FAD cofactor ( $\lambda_{max}$ around 450 nm), which is the origin of light-induced one-electron transfer that initiates radical-pair spin chemistry in photolyases and cryptochromes (Biskup et al., 2009; Sheppard et al., 2017). The precise role of 2 in this photolyase/cryptochrome subclade needs to be evaluated, in particular in its interplay with the FAD cofactor.

6,7-Dimethyl-8-ribityllumazine and certain structural analogs, e.g., 6,7,8-trimethyllumazine (3), exhibit anomalously high C–H acidity of the methyl group at position 7. For 2 and 3, p $K_{a}$ values of 8.3 (Pfleiderer et al., 1966; Bown et al., 1986) and 9.9 (Pfleiderer et al., 1966; McAndless and Stewart, 1970; Bown et al., 1986) have been reported, respectively. Using ${}^{1}$ H and ${}^{13}$ C NMR, compound 3 has been found to form an anionic species under alkaline conditions, which has been assigned a 7 α -exomethylene motif. Compound 2 forms additionally several tricyclic ether anion species under the participation of the OH groups of the ribityl side chain attached at position 8 (Bown et al., 1986). Interestingly, riboflavin synthase selectively binds the 7 α -exomethylene anion of 2, which is believed to be crucial for the dismutation of 2 affording a stoichiometric mixture of riboflavin (4) and 5-amino-6-ribitylaminouracil. This reaction has been shown to proceed via a pentacyclic intermediate which was isolated using an inactive mutant of riboflavin synthase (Illarionov et al., 2001). Various pathways have been proposed for the riboflavin synthesis from 2 (Truffault et al., 2001; Gerhardt et al., 2002; Kim et al., 2010). For the non-enzymatic reaction, a quantum mechanical simulation favors a nucleophilic addition mechanism, which was calculated as the lowest energy pathway yielding riboflavin (Breugst et al., 2013).

In this contribution, we report on a process between the neutral and the anionic 6,7,8-substituted lumazine species 3. Studies along these lines may ultimately shed light on the role of the related compound 2 in light-induced redox reactions of proteins from the CryPro subclade of photolyases and cryptochromes.

## 2 Experimental part

### 2.1 Sample preparation

6,7,8-Trimethyllumazine was prepared using a procedure described previously (Masuda, 1957) and purified using high-performance liquid chromatography (HPLC). For details, see the Supplement.

### 2.2 NMR and photo-CIDNP spectroscopy

NMR and photo-chemically induced dynamic nuclear polarization (photo-CIDNP) experiments were performed as described previously (Pompe et al., 2019), using a Bruker Avance III HD 600 MHz NMR spectrometer (Bruker BioSpin GmbH, Rheinstetten, Germany) operating at 14.1 T. Light excitation was achieved by coupling the output of a nanosecond-pulsed laser system, comprising an Nd:YAG laser source (Surelite I, Continuum, Santa Clara, CA, USA) in combination with a broadband optical parametric oscillator (OPO) (Continuum OPO PLUS), into an optical fiber with a diameter of 1 mm (Thorlabs, Dachau, Germany). The optical fiber was inserted into the NMR tube via a coaxial insert (Wilmad WGS-5BL). Photo-CIDNP difference spectra were recorded directly by using a pre-saturation pulse train to destroy thermal polarization prior to the laser flash (Goez et al., 2005). This avoids errors involved with the subtraction of light and dark spectra from separate experiments. A destructive phase cycle was additionally applied in which every second scan contained light excitation to avoid residual thermal NMR signals, especially contributions from the solvent peak (HDO) at 4.8 ppm.

### 2.3 Quantum chemical calculations

Molecular structures were drawn in Avogadro 1.2.0 (Hanwell et al., 2012) and were subsequently pre-optimized using the MMFF94 force field (Halgren, 1996b, a). Geometry optimizations were performed in Orca 4.0.1.2 (Neese, 2012, 2018) using the B3LYP functional (Stephens et al., 1994) and a TZVP basis set (Schäfer et al., 1994). The CPCM model (Barone and Cossi, 1998) was used to simulate water solvation. Calculations of hyperfine coupling constants and g matrices were then carried out by using a B3LYP functional together with the EPR-II basis set (Barone, 1996).

## 3 Results and discussions

**Figure 1:**
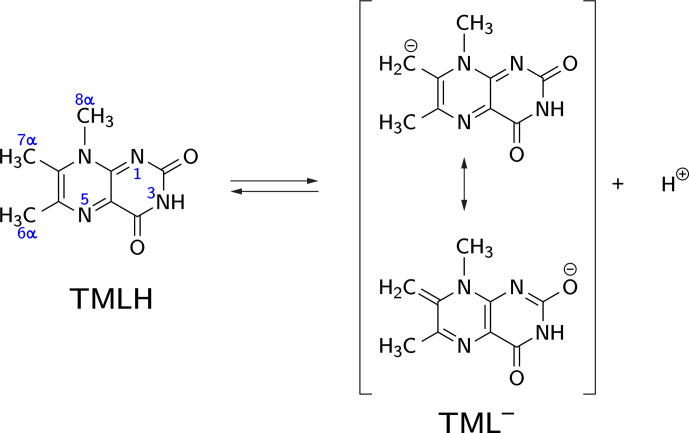
CH acidity of the methyl group attached to position 7 in 6,7,8-trimethyllumazine. The deprotonated (anionic) form TML ${}^{-}$ can be drawn in various mesomeric structures, two of which are depicted on the right-hand side.

**Figure 2:**
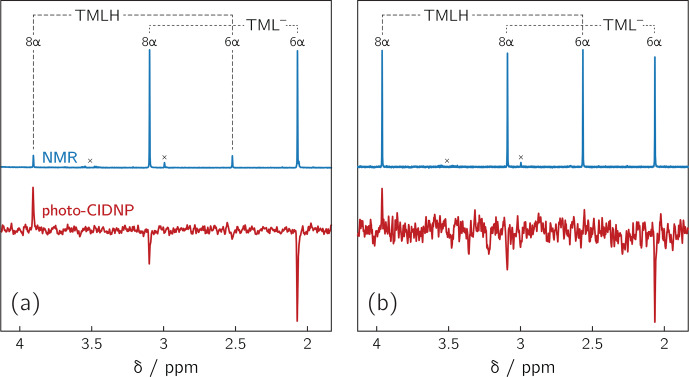
${}^{1}$ H NMR and photo-CIDNP data of alkaline solutions of 6,7,8-trimethyllumazine: **(a)** TMLH : TML ${}^{-}$ ratio of 1 : 10; 6,7,8-trimethyllumazine concentration: 4 mM; excitation wavelength: 425 nm; **(b)** TMLH : TML ${}^{-}$ ratio of 1 : 1; 6,7,8-trimethyllumazine concentration: 4 mM; excitation wavelength: 470 nm. Signals marked with “ × ” arise from sample impurities.

Deprotonation of 6,7,8-trimethyllumazine affords a structurally unusual 7 α -exomethylene anion (Beach and Plaut, 1970; Bown et al., 1986) subsequently designated TML ${}^{-}$ (see Fig. 1). With a reported p $K_{a}$ around 9.9 (Pfleiderer et al., 1966), 6,7,8-trimethyllumazine exhibits extraordinarily strong C–H acidity. For photo-CIDNP experiments, samples with a predetermined ratio of 6,7,8-trimethyllumazine (the neutral molecule form is subsequently designated TMLH; see Fig. 1) and the cognate anion designated TML ${}^{-}$ were prepared by the addition of NaOD to a 6,7,8-trimethyllumazine solution (1–4 mM range) in 99 % D ${}_{2}$ O. The TMLH : TML ${}^{-}$ ratio was monitored using ${}^{1}$ H NMR at 600 MHz. In 6,7,8-trimethyllumazine, the three methyl groups at positions 6, 7 and 8 and the hydrogen at N(3) afford ${}^{1}$ H resonances. In D ${}_{2}$ O, H(3) and the 7-methyl group exchange protons with the bulk solvent (Beach and Plaut, 1970); the latter exchange follows first-order kinetics on a double-digit minutes timescale (McAndless and Stewart, 1970). For the neutral TMLH species, we observed resonances of equal intensity at chemical shift values of 3.91 and 2.52 ppm, which we assigned as H(8 α ) and H(6 α ), respectively, based on reported values in the literature (4.02 ppm, H(8 α ); 2.61 ppm, H(6 α )) (McAndless and Stewart, 1970); see NMR spectra in Fig. 2. For TML ${}^{-}$ in alkaline solution, chemical shift values have been reported for H(8 α ) (3.15 ppm) and H(6 α ) (2.10 ppm) (Beach and Plaut, 1970). Accordingly, we assigned the resonances at 3.09 and 2.06 ppm to H(8 α ) and H(6 α ), respectively. Based on the integrals of the resonance lines of the two species, photo-CIDNP experiments were conducted at TML ${}^{-}$ -to-TMLH ratios of 1 : 11, 1 : 2, 1 : 1, 1.5 : 1, 7 : 1 and 10 : 1 at various pH values below, near or above the p $K_{a}$ .

**Figure 3:**
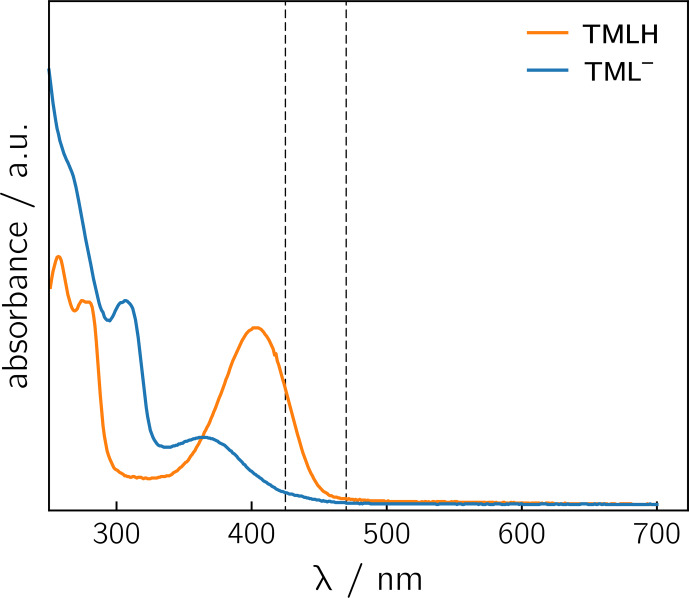
UV/vis spectra of TMLH and TML ${}^{-}$ recorded in water and in 1 M NaOH, respectively. The vertical dashed lines indicate the wavelengths chosen for sample irradiation (425 and 470 nm).

Photoexcitation of the TMLH / TML ${}^{-}$ solutions for ${}^{1}$ H photo-CIDNP experiments was performed using 6 ns pulses of an Nd:YAG-laser-pumped OPO adjusted to 425 or 470 nm (pulse energies of 8 mJ at 425 nm and 30 mJ at 470 nm). TMLH absorbs preferentially at these wavelengths because the long-wavelength absorbance of TML ${}^{-}$ (364 nm) is blue-shifted with respect to that of TMLH (402 nm) (Pfleiderer et al., 1966); see Fig. 3. Typically, 8 free induction decays (FIDs) were collected and averaged. Selected photo-CIDNP data are shown in Fig. 2. Three resonances exhibit substantial nuclear spin polarization: the position 6 and 8 methyl group signals of TML ${}^{-}$ and the position 8 methyl group signal of TMLH. The protons of the position 6 methyl group of TMLH do not show appreciable hyperpolarization. As in NMR, the position 7 methyl group does not exhibit any resonances in photo-CIDNP due to proton exchange with deuterons from the solvent.

**Figure 4:**
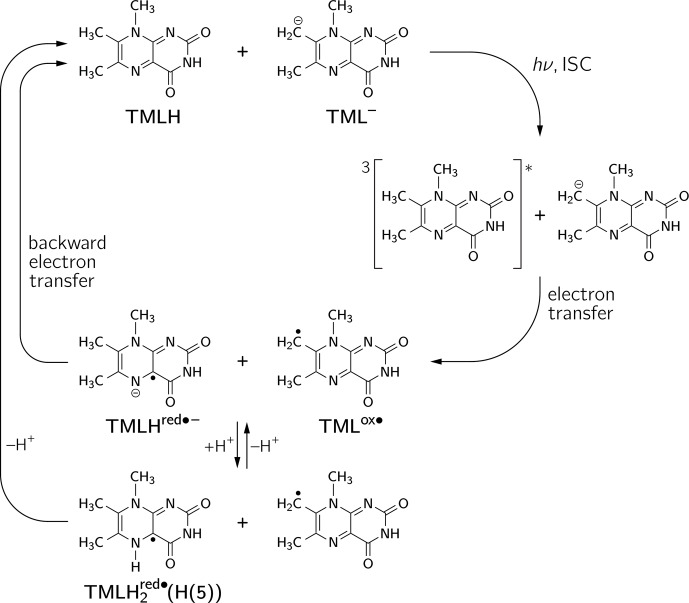
Photo-induced reversible disproportionation of 6,7,8-trimethyllumazine. ISC, intersystem crossing.

The occurrence of nuclear hyperpolarization upon photoexcitation of alkaline 6,7,8-trimethyllumazine samples provides clear evidence for a photochemical reaction involving radical pair intermediates. Since 6,7,8-trimethyllumazine is the only organic species present that absorbs light in the visible range, we suggest disproportionation to take place under the given conditions; see Fig. 4: light-initiated electron transfer from the anionic TML ${}^{-}$ to the neutral TMLH generates the neutral radical TML ${}^{\mathrm{ox}⚫}$ and initially the anionic radical TMLH ${}^{\mathrm{red}⚫-}$ as short-lived products. In the dark, backward electron transfer takes place to regenerate the initial species TML ${}^{-}$ and TMLH, respectively.

To corroborate this notion, we analyzed intensities and signs of the hyperpolarized NMR resonances. In the high-field approximation, the applied time-resolved photo-CIDNP scheme using a pulsed laser as a light source (Closs et al., 1985; Goez et al., 2005; Kuhn, 2013) renders signal intensities that are proportional to the isotropic hyperfine coupling constant $A_{\mathrm{iso}}$ of the respective nucleus (Adrian, 1971; Morozova et al., 2011). In the present case with only two weakly coupled ${}^{1}$ H spins per species, the relative enhancement factors can, in principle, be extracted by signal integration. Kaptein (1971) introduced a simple rule for the net polarization $\Gamma_{i}$ of a hyperpolarized resonance. $\Gamma_{i}$ results from the product of four signs and yields either “ + ” or “ - ” for an absorptive or an emissive signal, respectively:

1 $$\Gamma_{i}=\mu \times \varepsilon \times \mathrm{sgn}(\Delta g)\times \mathrm{sgn}(A_{\mathrm{iso},i}).$$

The parameter μ is either “ + ” in case the radical pair is formed from a triplet precursor or “ - ” in case of a singlet precursor. The reaction route following the formation of the intermediate radical pair determines the sign of ε , either “ + ” for recombination/re-encounter or “ - ” for dissociation, the latter leading to so-called escape products. The sign of the difference of the two (isotropic) g factors of the two involved radicals, $\Delta g=g_{1}-g_{2}$ , depends on which of the two radical moieties comprising the radical pair is observed (see below). Finally, the sign of the isotropic hyperfine coupling constant ( $A_{\mathrm{iso},i}$ ) of the respective nucleus i is of relevance.

To apply Kaptein's sign rule for rationalizing the polarization of a particular resonance in the photo-CIDNP spectrum of 6,7,8-trimethyllumazine, we consider the following aspects: (i) since the time delay between pulsed laser excitation and the radio-frequency pulse applied for the detection of the FID was chosen rather short ( ∼ 80 ns), the detection of recombination products is supposed to be more likely than that of escape products, thus ε→ “ + ”. (ii) Little is known about the g factors of paramagnetic lumazine species. Early EPR studies were focused on analyses of hyperfine patterns in EPR spectra from radicals of 6,7,8-trimethyllumazine (Ehrenberg et al., 1970) and derivatives thereof (Westerling et al., 1977), but they did not report on their g values. A more recent high-field EPR study considered 6,7-dimethyl-8-ribityllumazine bound to lumazine protein (Paulus et al., 2014). This cofactor was photo-reduced to yield the neutral 6,7-dimethyl-8-ribityllumazine radical, from which the isotropic g factor of 2.0032 ± 0.0001 was obtained by averaging the principal values of the g matrix. The lack of respective experimental data for the specific 6,7,8-trimethyllumazine radicals involved in disproportionation (see Fig. 4) prompted us to perform quantum-chemical computations at the density-functional theory (DFT) level to calculate the necessary values. Starting point for geometry optimization was a previously published structure model of 6,7,8-trimethyllumazine along with six coordinating water molecules (Schreier et al., 2011). Calculated $g_{\mathrm{iso}}$ values were 2.0031 for the neutral radical TML ${}^{\mathrm{ox}⚫}$ and 2.0034 for the anionic radical TMLH ${}^{\mathrm{red}⚫-}$ . Tentatively, the $g_{\mathrm{iso}}$ value of TMLH ${}^{\mathrm{red}⚫-}$ may be compared to the measured value of protein-bound 6,7-dimethyl-8-ribityllumazine radical because both species result from one-electron reduction of their aromatic moiety; nevertheless, the substituents bound to the 8-position and the protonation states of both radicals are different. Neglecting the unequal substitution at position 8, the neutral 6,7-dimethyl-8-ribityllumazine radical may be considered as the species obtained by protonation of TMLH ${}^{\mathrm{red}⚫-}$ to yield TMLH ${}_{2}^{\mathrm{red}⚫}$ (see Fig. 4). Extrapolated to the realm of the related flavins that share similar hydrogen-bonding motifs with the respective lumazines (see Scheme 1), the couple TMLH ${}^{\mathrm{red}⚫-}$ / TMLH ${}_{2}^{\mathrm{red}⚫}$ is expected to behave like anionic (Fl ${}^{⚫-}$ ) / neutral (FlH ${}^{⚫}$ ) flavin semiquinone radicals. For the latter, slightly larger $g_{\mathrm{iso}}$ values were observed for anion radicals than for neutral radicals (Schleicher and Weber, 2012): ∼ 2.0035 (Barquera et al., 2003; Okafuji et al., 2008) versus ∼ 2.0034 (Fuchs et al., 2002; Barquera et al., 2003; Schnegg et al., 2006), respectively, but the difference is quite small. This seems to also hold for the one-electron reduced lumazine radicals: $g_{\mathrm{iso}}$ (TMLH ${}^{\mathrm{red}⚫-}$ ) > $g_{\mathrm{iso}}$ (neutral 6,7-dimethyl-8-ribityllumazine radical). (iii) Absolute values of most proton hyperfine couplings have been determined for a cationic (one-electron reduced) 6,7,8-trimethyllumazine radical species (Ehrenberg et al., 1970) and for the neutral 6,7-dimethyl-8-ribityllumazine radical (Paulus et al., 2014). However, the signs of the $A_{\mathrm{iso}}$ values have not been determined experimentally. Since the available hyperfine data from the literature are only of limited value for the interpretation of our photo-CIDNP spectra, we performed quantum-chemical computations also of the hyperfine structure of the 6,7,8-trimethyllumazine radicals under discussion. The ${}^{1}$ H hyperfine couplings relevant for the interpretation of the photo-CIDNP NMR data are compiled in Table 1, together with the respective $g_{\mathrm{iso}}$ values. A full set of hyperfine couplings from all other protons as well as from ${}^{13}$ C and ${}^{15}$ N nuclei can be found in the Supplement (Tables S2 to S5). Additionally, the respective data for two further one-electron reduced species have been included that potentially result from protonation of the anionic TMLH ${}^{\mathrm{red}⚫-}$ at N(1) or N(5): TMLH ${}_{2}^{\mathrm{red}⚫}$ (H(1)) and TMLH ${}_{2}^{\mathrm{red}⚫}$ (H(5)), respectively. Importantly, for all one-electron reduced 6,7,8-trimethyllumazine radicals, DFT predicts isotropic hyperfine couplings of the H(8 α ) protons that are much larger than those of H(6 α ): $A_{\mathrm{iso}}$ (H(8 α )) ≫ $A_{\mathrm{iso}}$ (H(6 α )) (see Table 1); EPR data are consistent with this finding (Ehrenberg et al., 1970). For the TML ${}^{\mathrm{ox}⚫}$ radical that results from TML ${}^{-}$ by withdrawal of one electron, DFT predicts $A_{\mathrm{iso}}$ (H(6 α )) > $A_{\mathrm{iso}}$ (H(8 α )).

**Table 1:** Isotropic g values and selected isotropic methyl proton hyperfine couplings for various oxidized and reduced 6,7,8-trimethyllumazine radicals.

		TMLH ${}^{\mathrm{red}⚫-}$		TMLH ${}_{2}^{\mathrm{red}⚫}$ (H(5))		TMLH ${}_{2}^{\mathrm{red}⚫}$ (H(1))		TML ${}^{\mathrm{ox}⚫}$	
$g_{\mathrm{iso}}$ (DFT)		2.0034		2.0033		2.0034		2.0031	
Proton		6 α	8 α	6 α	8 α	6 α	8 α	6 α	8 α
$A_{\mathrm{iso}}$ (DFT)	abs./MHz	-5.31	+15.21	+1.57	+18.90	-5.38	+14.41	+14.93	+6.42
	rel.	-0.165	0.473	0.039	0.473	-0.177	0.473	1	0.430
$A_{\mathrm{iso}}$ (CIDNP)	rel.	0	0.473	0	0.473	0	0.473	1	0.439

Photo-excitation of an alkaline 6,7,8-trimethyllumazine solution (4 mM; TMLH : TML ${}^{-}$ ratio of 1 : 10) with 425 nm laser pulses resulted in a photo-CIDNP spectrum with both TML ${}^{-}$ resonances in emission (see Fig. 2a). The ratio of the integrals of the signals assigned to H(6 α ) and H(8 α ) is 1 : 0.439. The situation is different for the signals assigned to TMLH: whereas the H(8 α ) resonance exhibits enhanced absorption, the one of H(6 α ) does not show significant hyperpolarization. Virtually the same polarization pattern is observed for a less alkaline 6,7,8-trimethyllumazine solution (4 mM) with a TMLH : TML ${}^{-}$ ratio of 1 : 1, i.e., pH ≈ p $K_{a}$ (see Fig. 2b). However, to obtain a discernible photo-CIDNP spectrum in this case, the excitation wavelength of our laser system had to be tuned to 470 nm. The higher wavelength was useful because of the rather high TMLH concentration and the high absorbance associated therewith, which did not allow for sufficient photo-excitation of the active sample volume given the available output power of our laser source at 425 nm. Nevertheless, the obtained signal-to-noise ratio remained rather low and it even decreased upon further decreasing the amount of TML ${}^{-}$ relative to that of TMLH. Observation of the photo-CIDNP effect at 470 nm is clear evidence for photo-excitation of TMLH rather than TML ${}^{-}$ . The latter has very low absorbance at 425 nm and does not virtually absorb at 470 nm (Pfleiderer et al., 1966; see Fig. 3).

By far the highest signal-to-noise ratio of the photo-CIDNP data was obtained in the alkaline range at about one pH unit above the p $K_{a}$ of TMLH / TML ${}^{-}$ . We could not conduct NMR experiments under more basic pH conditions because the high ion strength of our sample precluded proper tuning of the probe head. Additionally, we varied the 6,7,8-trimethyllumazine concentration in a range between 1.0 and 4.0 mM. In all cases, photo-CIDNP revealed a hyperpolarization pattern similar to the one shown in Fig. 2a.

**Figure 5:**
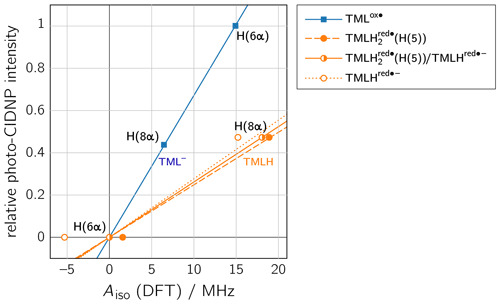
Correlations of relative photo-CIDNP intensities to predicted $A_{\mathrm{iso}}$ values from DFT. TML ${}^{\mathrm{ox}⚫}$ : slope = 0.0672 MHz ${}^{-1}$ , $R^{2}$ = 0.9996; TMLH ${}^{\mathrm{red}⚫-}$ : slope = 0.0277 MHz ${}^{-1}$ , $R^{2}$ = 0.7827; TMLH ${}_{2}^{\mathrm{red}⚫}$ (H(5)): slope = 0.0248 MHz ${}^{-1}$ , $R^{2}$ = 0.9863; linear combination of TMLH ${}_{2}^{\mathrm{red}⚫}$ (H(5)) and TMLH ${}^{\mathrm{red}⚫-}$ with a ratio of 0.772 : 0.228: slope = 0.0262 MHz ${}^{-1}$ , $R^{2}$ = 1. CIDNP intensities were multiplied by the respective sgn(Δg) .

To rationalize our findings, we correlated the relative intensities of the hyperpolarized NMR resonances obtained by photo-CIDNP to DFT predictions of hyperfine couplings of the various paramagnetic one-electron oxidized or reduced lumazine species involved in the suggested disproportionation scheme along a procedure introduced by Ivanov and Yurkovskaya (Morozova et al., 2011; see Table 1, Fig. 5 and Supplement). Upon backward electron transfer in the dark, i.e., radical pair recombination ( ε = “ + ”), hyperpolarization generated on the intermediate oxidized species TML ${}^{\mathrm{ox}⚫}$ and the reduced species TMLH ${}^{\mathrm{red}⚫-}$ (or a protonated neutral species TMLH ${}_{2}^{\mathrm{red}⚫}$ thereof) is transferred to the diamagnetic products TML ${}^{-}$ and TMLH, respectively.

Considering the hyperpolarized NMR resonances of TML ${}^{-}$ leads to $\Delta g=g({\mathrm{TML}}^{\mathrm{ox}⚫})-g({\mathrm{TMLH}}^{\mathrm{red}⚫-})<0$ (see Table 1); i.e., the sign entering Eq. (1) becomes negative: sgn(Δg)= “ - ”. Furthermore, the hyperfine couplings from the precursor state TML ${}^{\mathrm{ox}⚫}$ are relevant. The calculated isotropic H(6 α ) and H(8 α ) hyperfine couplings of TML ${}^{\mathrm{ox}⚫}$ are positive; hence $\mathrm{sgn}(A_{\mathrm{iso},i})=$ “ + ” for i∈(H(6α),H(8α)) . Given the fact that both resonances are emissively polarized, i.e., $\Gamma_{i}$ = “ - ”, requires μ = “ + ” for the precursor multiplicity. Consequently, radical pair formation must proceed from a triplet-state precursor of radical pair formation, i.e., from ${}^{3}$ TMLH, which is generated by intersystem crossing from an excited singlet state ( ${}^{1*}$ TMLH) of TMLH (see Fig. 4). Plotting the photo-CIDNP intensities with respect to the hyperfine couplings obtained using DFT (see Fig. 5) reveals nearly perfect correlation: a linear regression fit constrained to go through the origin yields a slope of 0.0672 MHz ${}^{-1}$ and $R^{2}$ = 0.9996. Observation of hyperpolarized resonances of H(6 α ) and H(8 α ) from TML ${}^{-}$ both in emission and in an intensity ratio that correlates well with hyperfine coupling computations from DFT provides clear evidence for the existence of the oxidized TML species TML ${}^{\mathrm{ox}⚫}$ , which is a redox state of lumazine that up to now had not been substantiated by experiments.

If we retain the signs of ε (“ + ”, recombination) and μ (“ + ”, triplet precursor) and reverse the sign of Δg to “ + ” (because $\Delta g=g({\mathrm{TML}}^{\mathrm{red}⚫-})-g({\mathrm{TML}}^{\mathrm{ox}⚫})>0$ ), then we expect for the H(8 α ) resonance of TMLH because of $\Gamma_{i}$ = “ + ” (absorptive resonance) a positive isotropic hyperfine coupling constant of its paramagnetic precursor state. A very small hyperfine coupling near or equal to zero is expected for H(6 α ) as hardly any hyperpolarization is observed for these nuclei in the photo-CIDNP spectrum (Fig. 2). This situation is reversed as compared to that of TML ${}^{-}$ , for which the H(6 α ) resonance experiences much stronger hyperpolarization than H(8 α ). Our DFT calculations confirm a large and positive value for $A_{\mathrm{iso}}$ (H(8 α )) of TMLH ${}^{\mathrm{red}⚫-}$ but also predict a negative hyperfine coupling of substantial absolute value for $A_{\mathrm{iso}}$ (H(6 α )). This latter finding is clearly not supported by the photo-CIDNP data shown in Fig. 2. Therefore, we have extended our DFT studies of 6,7,8-trimethyllumazine radicals to protonated variants of TMLH ${}^{\mathrm{red}⚫-}$ , namely the neutral species TMLH ${}_{2}^{\mathrm{red}⚫}$ (H(1)) (protonated at N(1)) and TMLH ${}_{2}^{\mathrm{red}⚫}$ (H(5)) (protonated at N(5)); see Tables 1, S3 and S4.

Protonation of TMLH ${}^{\mathrm{red}⚫-}$ at N(1) to yield TMLH ${}_{2}^{\mathrm{red}⚫}$ (H(1)) does not significantly alter the isotropic hyperfine couplings of H(6 α ) and H(8 α ); also $g_{\mathrm{iso}}$ is virtually unaffected (see Tables 1 and S4). However, addition of a proton at N(5) to yield TMLH ${}_{2}^{\mathrm{red}⚫}$ (H(5)) does shift both hyperfine couplings to more positive values. $A_{\mathrm{iso}}$ (H(6 α )) even changes its sign and assumes a small positive value, but more than 10 times smaller than that of $A_{\mathrm{iso}}$ (H(8 α )); see Tables 1 and S3. Our photo-CIDNP data thus support rapid protonation of TMLH ${}^{\mathrm{red}⚫-}$ at N(5) to yield the neutral radical species TMLH ${}_{2}^{\mathrm{red}⚫}$ (H(5)). When considering a linear combination of the hyperfine data of TMLH ${}_{2}^{\mathrm{red}⚫}$ (H(5)) and TMLH ${}^{\mathrm{red}⚫-}$ (see TMLH ${}_{2}^{\mathrm{red}⚫}(H(5))/{\mathrm{TMLH}}^{\mathrm{red}⚫-}$ in Fig. 5) with a ratio of 0.772 : 0.228, we obtain $A_{\mathrm{iso}}$ (H(6 α )) = 0 and $A_{\mathrm{iso}}$ (H(8 α )) = 18.1 MHz and consequently, of course, perfect correlation of DFT-calculated hyperfine couplings and relative photo-CIDNP intensities with $R^{2}$ = 1 and a slope of 0.0262 MHz ${}^{-1}$ . Such an approach has been successfully applied previously to photo-CIDNP studies of other systems; see, e.g., Morozova et al. (2018) and Torres et al. (2021). The obtained ratio of TMLH ${}_{2}^{\mathrm{red}⚫}$ (H(5)) to TMLH ${}^{\mathrm{red}⚫-}$ should be treated with caution as the photo-CIDNP intensities were correlated with hyperfine data predicted from DFT because experimental values are unavailable. The accuracy of DFT hyperfine predictions however strongly depends on the choice of functional basis set and molecular geometry (Kirste, 2016; Witwicki et al., 2020).

The slopes of the straight lines through the origin in the correlation plots of TML ${}^{-}$ and TMLH in Fig. 5 are clearly different: 0.0672 MHz ${}^{-1}$ versus 0.0262 MHz ${}^{-1}$ (for TMLH ${}_{2}^{\mathrm{red}⚫}(H(5))/{\mathrm{TMLH}}^{\mathrm{red}⚫-}$ ), respectively. Furthermore, even though $A_{\mathrm{iso}}$ of H(8 α ) in TMLH ${}_{2}^{\mathrm{red}⚫}$ has a larger value than $A_{\mathrm{iso}}$ of H(6 α ) in TML ${}^{\mathrm{ox}⚫}$ , the corresponding photo-CIDNP intensity of the resonance in the recombination product is significantly smaller than that of the most intense signal of the respective counter radical. These findings may have several reasons: (i) introduction of a proton at N(5) adds a further large hyperfine coupling ( $A_{\mathrm{iso}}$ (H(5)) of substantial anisotropy (see Table S3). Protonation dynamics of H(5) could enhance relaxation by which hyperpolarized spin-state population decays to the population at thermal equilibrium. (ii) Hyperpolarization could also be dissipated into the solvent upon radical-pair recombination. Backward electron transfer from TMLH ${}_{2}^{\mathrm{red}⚫}$ yields the diamagnetic TMLH ${}_{2}^{+}$ , a species that will certainly deprotonate quickly to regenerate TMLH, especially given the alkaline conditions. Hence, intermediate electron-spin redistribution leading to buildup of hyperpolarization at H(5) will likely be transferred to the surroundings on release of this proton. (iii) Despite the fact that electron exchange has been observed in other systems leading to a decay of hyperpolarization (Closs and Sitzmann, 1981), we consider such a mechanism along the scheme ${}^{\#}{\mathrm{TMLH}}^{\mathrm{red}⚫-}+\mathrm{TMLH}\to^{\#}\mathrm{TMLH}+{\mathrm{TMLH}}^{\mathrm{red}⚫-}$ (“ ${}^{\#}$ ” denotes nuclear spin polarization) less likely given that at the elevated pH values under consideration neutral TMLH is present only at rather low concentrations.

**Figure 6:**
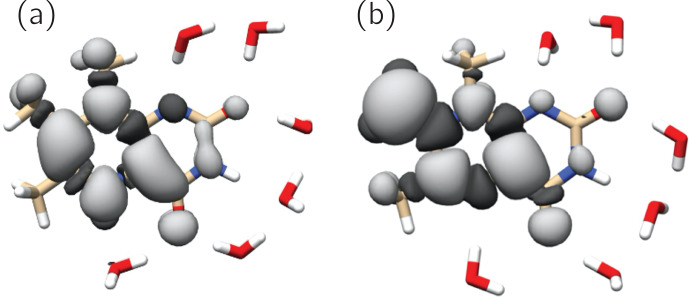
SOMOs of observed 6,7,8-trimethyllumazine radical species. Dark and light grey contours denote negative and positive signs of the molecular wavefunctions. **(a)** TMLH ${}_{2}^{\mathrm{red}⚫}$ (H(5)); **(b)** TML ${}^{\mathrm{ox}⚫}$ .

Figure 6 shows the singly occupied molecular orbitals (SOMOs) of the neutral radicals TMLH ${}_{2}^{\mathrm{red}⚫}$ (H(5)) and TML ${}^{\mathrm{ox}⚫}$ . Positive and negative signs of the frontier orbitals are depicted with light and dark grey shading, respectively. Mere inspection gives a hint for the quite different ratios of isotropic hyperfine couplings of H(8 α ) and H(6 α ) in both species: the amplitude of the SOMO at the 6-methyl group of TMLH ${}_{2}^{\mathrm{red}⚫}$ (H(5)) is small as compared to that of TML ${}^{\mathrm{ox}⚫}$ . For the 8-methyl group the opposite trend is observed. Considerable SOMO amplitudes are observed for H(5) in TMLH ${}_{2}^{\mathrm{red}⚫}$ (H(5)), which leads to a strong and anisotropic hyperfine coupling of this exchangeable proton. This explains the dissipation of hyperpolarization into the solvent on backward electron transfer leading from TMLH ${}_{2}^{\mathrm{red}⚫}$ (H(5)) to TMLH and H ${}^{+}$ .

The SOMOs of TMLH ${}_{2}^{\mathrm{red}⚫}$ (H(5)) and TML ${}^{\mathrm{ox}⚫}$ significantly differ in terms of wavefunction amplitudes and signs, in particular at the respective π system of the pyrazine ring. Very high amplitude is observed at C(7 α ) of TML ${}^{\mathrm{ox}⚫}$ . This gives a hint that the electronic structure of the related one-electron reduced TML ${}^{-}$ may be better represented by a C(7 α )-carbanion rather than a 7 α -exomethylene motif at position 7 (see Fig. 1). Clearly, the two observable proton resonances per radical species and their hyperpolarization detected using photo-CIDNP NMR are insufficient to draw a precise picture of the delocalization of the unpaired electron spin density over the carbons and nitrogens in the heterocyclic cores. To learn more about the electron-spin distributions in the two radicals generated by disproportionation of 3 and to further corroborate the existence of the oxidized species TML ${}^{\mathrm{ox}⚫}$ , we plan further photo-CIDNP experiments on specifically designed ${}^{13}$ C and ${}^{15}$ N isotopologs of 2 and 3.

## 4 Conclusions

Using photo-CIDNP NMR we have discovered a disproportionation reaction upon photoexcitation of alkaline solutions of 6,7,8-trimethyllumazine. In its classical definition, disproportionation refers to a “reversible or irreversible transition in which species with the same oxidation state combine to yield one of higher oxidation state and one of lower oxidation state” (McNaught and Wilkinson, 1997). This includes redox reactions of the following type: $2X\to X^{\mathrm{ox}⚫+}+X^{\mathrm{red}⚫-}$ . From the perspective of oxidation states, this scheme applies to 6,7,8-trimethyllumazine, which upon photo-induced electron transfer generates a pair of radicals comprising a species devoid of one electron (TML ${}^{\mathrm{ox}⚫}$ ) and another with an excess electron (TMLH ${}^{\mathrm{red}⚫-}$ ). However, our proposed mechanism deviates from the classical disproportionation scheme in so far as (i) the reaction is initiated *directly* by light and (ii) that a redox reaction takes place between two *different* protonation states of 6,7,8-trimethyllumazine, i.e., between TMLH and TML ${}^{-}$ . Clearly, disproportionation starts out from photoexcitation of TMLH (notably, only TMLH has significant absorption at 470 nm). Once the triplet state of TMLH is formed by intersystem crossing from an excited singlet state of this molecule, it abstracts an electron from TML ${}^{-}$ , thereby generating a pair of interacting radicals (see Fig. 4). Interestingly, one quite unusual radical species is generated by this disproportionation that has not been reported before: TML ${}^{\mathrm{ox}⚫}$ . The existence of a species in such a high redox state was speculated upon in triplet quenching of the unsubstituted lumazine (1) (Denofrio et al., 2012); one should however keep in mind that the aromatic moiety of 6,7,8-trimethyllumazine (3) differs from that of the unsubstituted lumazine. A similar species was proposed for triplet quenching of the related flavins (Görner, 2007), but convincing experimental evidence on the existence of a species in such a high oxidation state was still lacking for both cases until now. By detecting two important hyperfine couplings, we provide strong evidence for the existence of 6,7,8-trimethyllumazine in a further high redox state. Clearly, we owe this success to two peculiarities of 6,7,8-trimethyllumazine: (i) the extraordinary acidity of its 7-methyl group which compares to that of the ammonium ion and (ii) its proton exchange on a timescale that is slow compared to that of NMR and which consequently leads to distinguishable anionic (TML ${}^{-}$ ) and neutral (TMLH) protonation forms in terms of NMR properties. Flavins by comparison do not exhibit a corresponding acidity of their methyl groups. Therefore, our photo-CIDNP detection scheme is not readily extendable to the realm of flavins for a proof of the existence of the speculative FAD ${}^{\mathrm{ox}⚫+}$ species.

Our data on 6,7,8-trimethyllumazine provide evidence for an extended range of redox states of lumazines in general. Further studies on lumazine-mediated photocatalysis will show whether the existence of species of the type of TML ${}^{\mathrm{ox}⚫}$ will be involved that shed new light on the role of 6,7-dimethyl-8-ribityllumazine as chromophore, e.g., in the new class of recently discovered CryB cryptochromes (Geisselbrecht et al., 2012) and PhrB photolyases (Oberpichler et al., 2011; Zhang et al., 2013) of the CryPro subclade. Further studies will be conducted on the suitability of TML as a photosensitizer.

## Supplement

10.5194/mr-2-281-2021-supplement The supplement related to this article is available online at: https://doi.org/10.5194/mr-2-281-2021-supplement.

## Data Availability

Raw data are available at https://freidok.uni-freiburg.de/data/194780 (Wörner et al., 2021).

## Code availability

For information regarding the correlation plot, please contact the corresponding author.
