# Supplementary material for: Non-classical disproportionation revealed by photo-chemically induced dynamic nuclear polarization NMR
Source: Magn Reson (Gott). 2021 May 7;2(1):281–90. doi: 10.5194/mr-2-281-2021 (PMC10539781; doi:10.5194/mr-2-281-2021)
Supplement: The supplement related to this article is available online at: https://doi.org/10.5194/mr-2-281-2021-supplement. [file mr-2-281-supplement.pdf]

Supplement of Magn. Reson., 2, 281–290, 2021
https://doi.org/10.5194/mr-2-281-2021-supplement
© Author(s) 2021. CC BY 4.0 License.

Supplement of

Non-classical disproportionation revealed by photo-chemically induced dynamic nuclear polarization NMR

Jakob Wörner et al.

Correspondence to: Stefan Weber (stefan.weber@physchem.uni-freiburg.de)

The copyright of individual parts of the supplement might differ from the article licence.

Preparation of 6,7,8-trimethyllumazine

6,7,8-Trimethyllumazine was synthesized along a route described previously starting out with the preparation of 6-methyl-aminouracil (Masuda, 1957).

6-Methylaminouracil. A mixture of 6-chlorouracil (3 mmol, 0.44 g) and methylamine (40 wt. % in H2O, 5 ml) was stirred under reflux overnight. The white solid (0.37 g, 88%) was harvested by filtration, washed with water (5 ml), methanol (10 ml), and Et_2O (20 ml), and dried over P_2O_5; ^{1}H NMR (DMSO-6d, 400 MHz): δ [ppm] = 10.11–10.07 (m, 2H), 6.03–6.01 (m, 1H), 4.35 (s, 1H), 2.64 (d, 3H, J = 1.22 Hz); ^{13}C NMR (DMSO-6d, 100 MHz): δ [ppm] = 164.1, 154.9, 150.8, 72.3, 28.3; HRMS (ESI): *m*/*z* for $C_5H_6O_2N_3$ [M-H]$^-$ calculated: 140.0485, found: 140.0466.

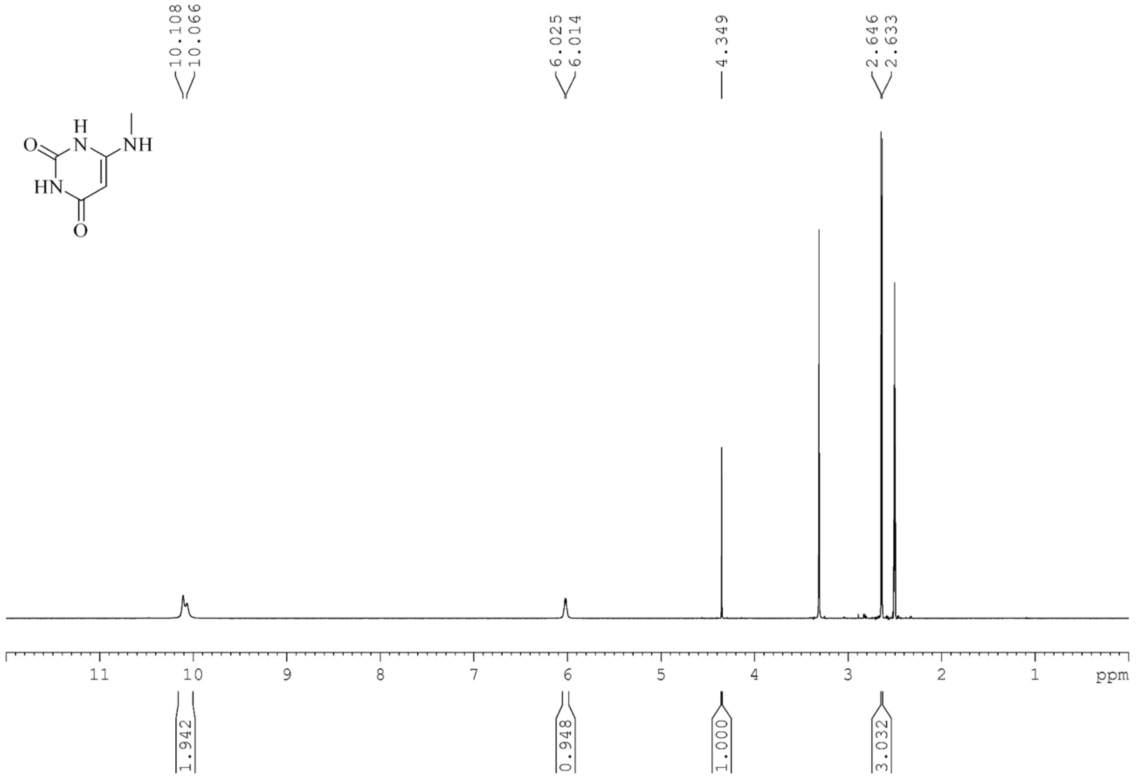

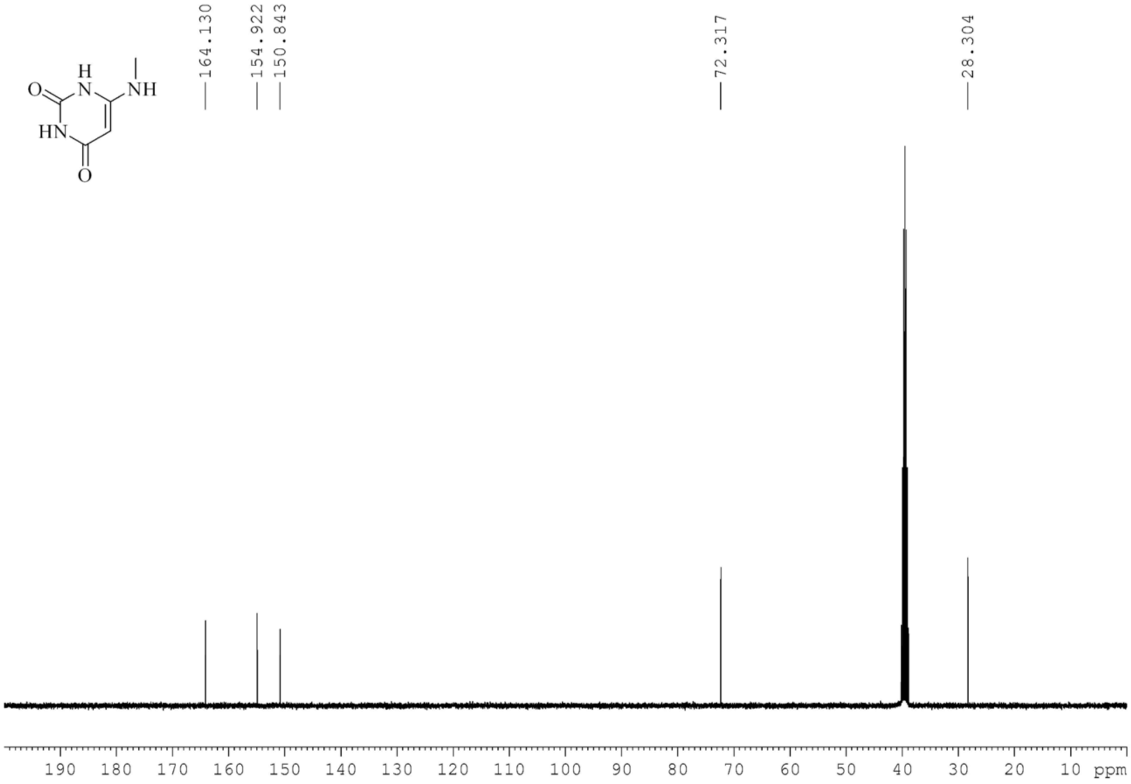
O
H
N
NH
HN
O
164.130
154.922
150.843
72.317
28.304
190 180 170 160 150 140 130 120 110 100 90 80 70 60 50 40 30 20 10 ppm

5-Nitroso-6-methylaminouracil. 6-Methylaminouracil (2.5 mmol, 0.35 g) was suspended in 6 ml of water, and $NaNO_2$ (0.35 g, 5 mmol) was added. Acetic acid (3.5 M) was added slowly until pH 4 was reached. Then the mixture was stirred for 30 min at room temperature. A red solid (0.39 g, 92%) was obtained by filtration, washed with water, and dried over P_2O_5; ^{1}H NMR (DMSO-6d, 400 MHz): δ [ppm] = 11.18, 2.95 (d, 3H, J = 1.07 Hz); ^{13}C NMR (DMSO-6d, 100 MHz): δ [ppm] = 160.7, 28.2.

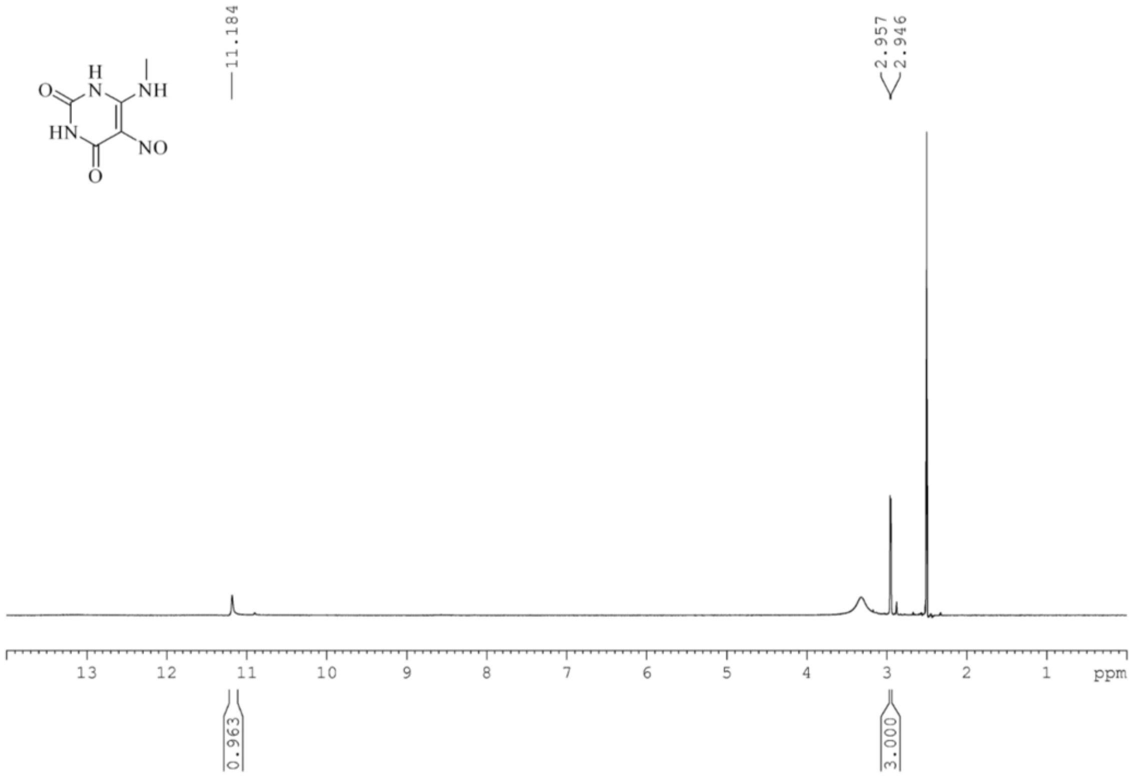

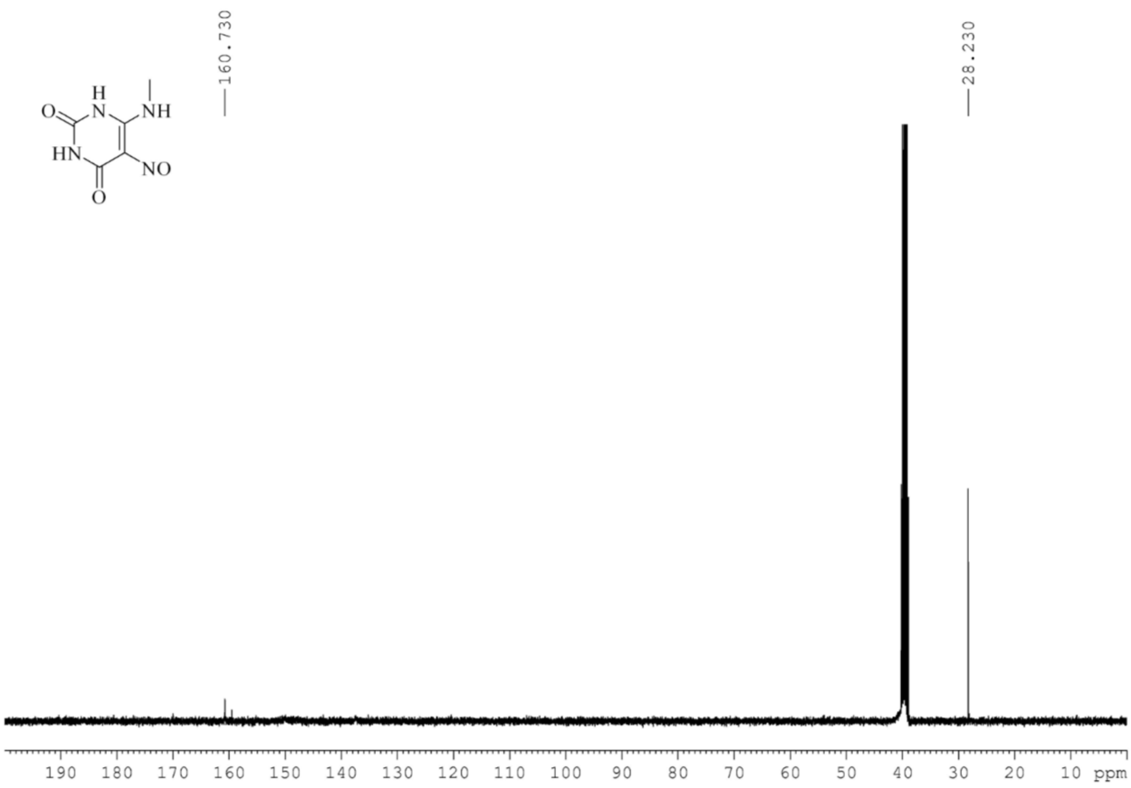

6,7,8-Trimethyllumazine. 5-Nitroso-6-methylaminouracil (0.34 g, 2 mmol) was suspended in 7 ml of water, and the mixture was heated to 100 °C. $Na_2S_2O_4$ was added in portions until the color of the solution changed from red to yellow. Butane-2,3-dione (0.77 ml) was added, and the mixture was stirred for 20 min at 75 °C. The mixture was then kept at 0 °C. A yellow solid (0.15 g, 36.4 %) was obtained by filtration, washed with cold methanol and Et_2O, and dried over P_2O_5; ^{1}H NMR (DMSO-6d, 600 MHz): δ [ppm] = 11.02 (s, 1H), 3.88 (s, 3H), 2.62 (s, 3H); ^{13}C NMR (DMSO-6d, 150 MHz): δ [ppm] = 160.8, 155.4, 151.1, 147.8, 139.1, 131.6, 34.9, 21.4, 17.6; HRMS (ESI): *m/z* for $C_9H_{11}O_2N_4$ $[M+H]^+$ calculated: 207.0877, found: 207.0876.

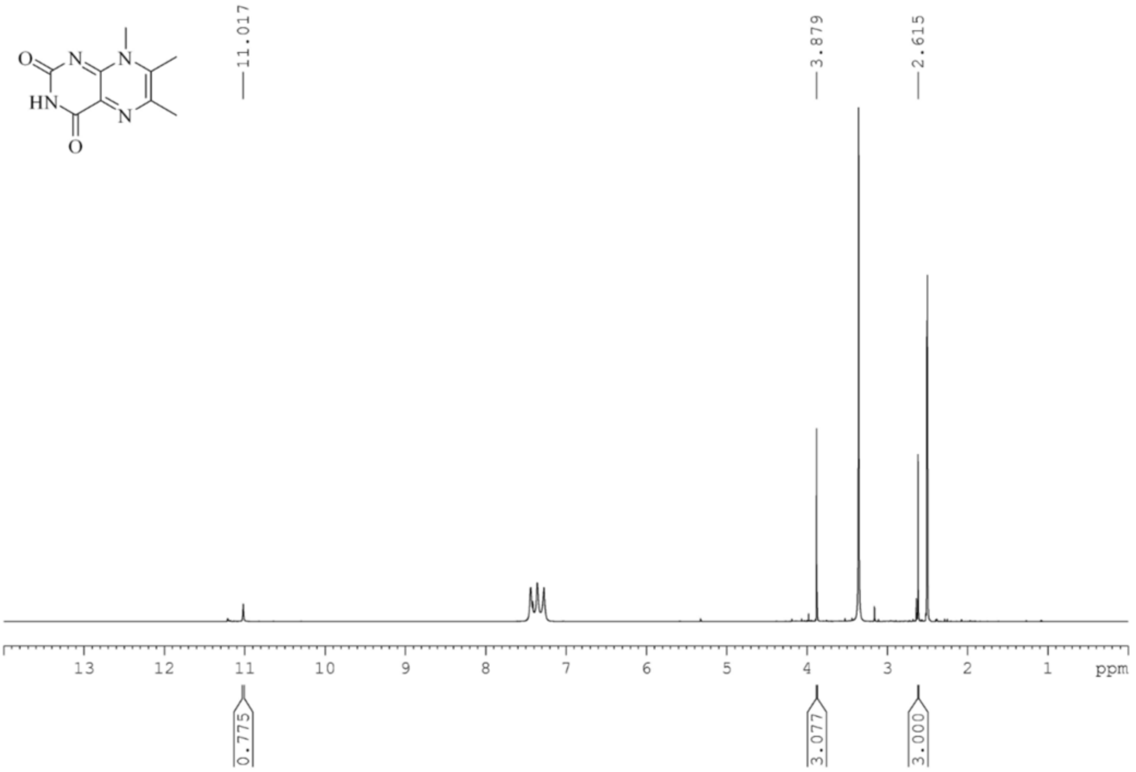

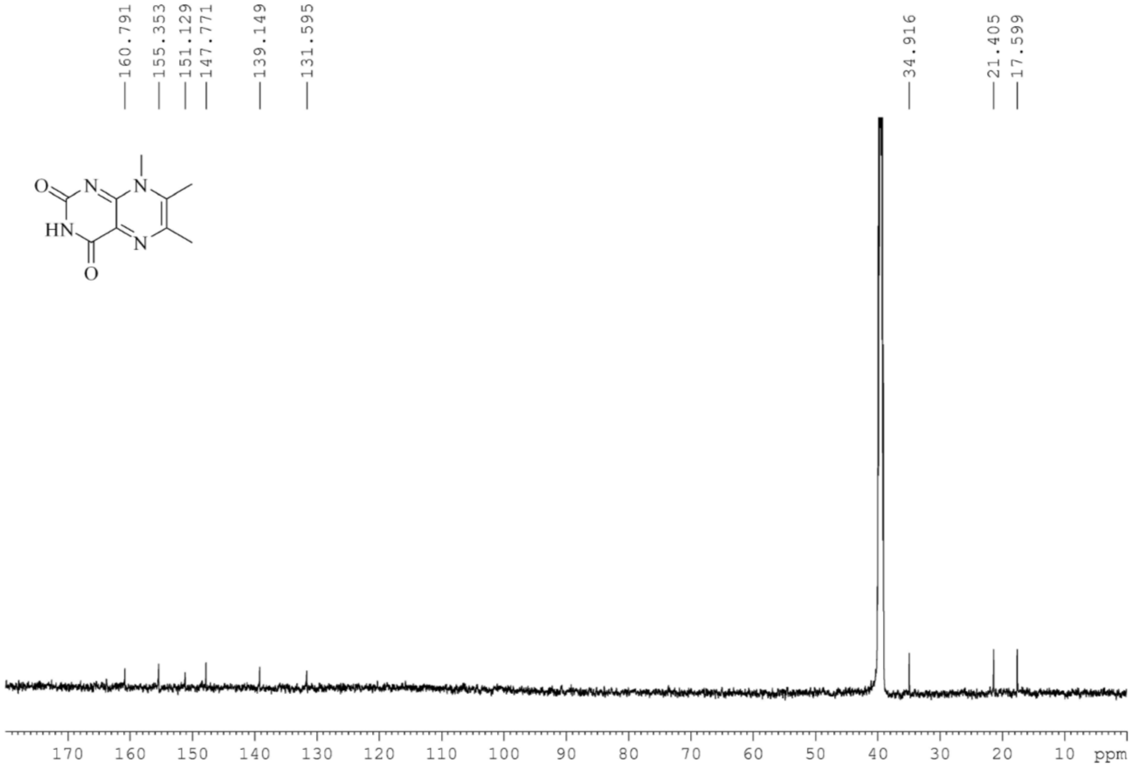
160.791
155.353
151.129
147.771
139.149
131.595
34.916
21.405
17.599
O
N
N
HN
N
O
170 160 150 140 130 120 110 100 90 80 70 60 50 40 30 20 10 ppm

Magnetic-resonance parameters obtained from density functional theory

Table S1: Principal values and isotropic g-values for various oxidized and reduced TML radicals.

	g_{XX}	g_{YY}	g_{ZZ}	g_{iso}
$TMLH^{red\bullet-}$	2.00221	2.00377	2.00421	2.00340
$TMLH_2^{red\bullet}$(H(5))	2.00222	2.00343	2.00419	2.00328
$TMLH_2^{red\bullet}$(H(1))	2.00223	2.00383	2.00422	2.00342
$TML^{ox\bullet}$	2.00235	2.00316	2.00372	2.00308

Table S2: Hyperfine couplings of $TMLH^{red\bullet-}$ in MHz.

atom	A_{XX}	A_{YY}	A_{ZZ}	A_{iso}
N(1)	0.058	–0.152	–2.105	–0.733
C(2)	–0.216	–1.137	2.766	0.471
O(2)	0.598	0.739	–4.501	–1.055
N(3)	0.448	–0.987	–1.238	–0.593
H(3)	1.181	–2.197	–2.496	–1.170
C(4)	–6.275	–7.695	13.813	–0.052
O(4)	3.460	3.983	–21.205	–4.587
C(4a)	2.839	–14.542	–16.874	–9.526
N(5)	–0.743	–0.953	50.019	16.108
C(6)	–20.257	–24.640	–39.781	–28.226
C(6α)	0.152	0.506	1.420	0.692
H(6α)	–3.461	–7.306	–8.136	–6.301
H(6α)	–5.281	–8.843	–9.816	–7.980
H(6α)	1.783	–3.134	–3.585	–1.645
C(7)	–2.181	–2.841	86.224	27.067
C(7α)	–13.314	–14.182	–15.089	–14.195
H(7α)	0.838	1.236	7.755	3.276
H(7α)	49.406	49.668	56.044	51.706
H(7α)	29.148	30.011	35.893	31.684
N(8)	–1.142	–1.394	28.585	8.683
C(8α)	–6.495	–8.181	–8.401	–7.692
H(8α)	7.543	8.394	14.070	10.002
H(8α)	3.541	3.764	9.419	5.575
H(8α)	28.043	28.269	33.892	30.068
C(8a)	–8.331	–9.948	–13.709	–10.663

Table S3: Hyperfine couplings of $TMLH_2^{red\bullet}$(H(5)) in MHz.

atom	A_{XX}	A_{YY}	A_{ZZ}	A_{iso}
N(1)	–0.052	0.190	–2.310	–0.724
C(2)	–0.377	0.505	–1.409	–0.427
O(2)	0.528	0.711	–3.955	–0.905
N(3)	–0.687	–0.995	1.697	0.005
H(3)	1.014	–3.094	–3.201	–1.760
C(4)	4.974	–8.136	–9.990	–4.384
O(4)	3.479	3.849	–20.170	–4.281
C(4a)	–12.725	–14.401	24.858	–0.756
N(5)	–0.915	–1.221	44.747	14.204
H(5)	–1.964	–30.261	–43.507	–25.244
C(6)	–9.055	–17.334	–19.952	–15.447
C(6α)	–0.736	–1.629	–1.986	–1.450
H(6α)	0.571	1.383	5.843	2.599
H(6α)	1.419	2.312	6.501	3.411
H(6α)	2.091	–2.743	–3.288	–1.313
C(7)	–8.226	–8.867	54.308	12.405
C(7α)	–9.026	–9.635	–10.616	–9.759
H(7α)	0.039	0.323	6.327	2.230
H(7α)	36.595	36.894	42.648	38.712
H(7α)	21.039	22.088	27.164	23.430
N(8)	–0.069	–0.392	34.395	11.311
C(8α)	–7.542	–8.615	–9.478	–8.545
H(8α)	11.083	12.121	17.650	13.618
H(8α)	3.991	4.313	10.185	6.163
H(8α)	34.827	35.036	40.919	36.927
C(8a)	–11.124	–12.891	–19.000	–14.338

Table S4: Hyperfine couplings of $TMLH_2^{red\bullet}$(H(1)) in MHz.

atom	A_{XX}	A_{YY}	A_{ZZ}	A_{iso}
N(1)	–0.048	0.152	–1.815	–0.570
H(1)	–0.817	–1.227	2.738	0.231
C(2)	–0.003	–0.904	4.051	1.048
O(2)	0.861	0.960	–5.818	–1.332
N(3)	–0.709	–1.019	–1.237	–0.988
H(3)	–1.141	1.294	–1.769	–0.539
C(4)	–5.621	–6.919	11.529	–0.337
O(4)	3.637	4.183	–21.161	–4.447
C(4a)	0.911	–14.200	–16.659	–9.983
N(5)	–0.914	–1.145	50.909	16.283
C(6)	–20.607	–25.140	–40.620	–28.789
C(6α)	0.417	0.878	1.842	1.046
H(6α)	–3.070	–7.161	–7.965	–6.065
H(6α)	–5.455	–9.060	–10.086	–8.200
H(6α)	1.600	–3.349	–3.834	–1.861
C(7)	–1.273	–2.106	93.036	29.886
C(7α)	–13.686	–14.535	–15.586	–14.602
H(7α)	2.183	2.627	9.374	4.728
H(7α)	52.044	52.242	58.968	54.418
H(7α)	28.591	29.563	35.580	31.245
N(8)	–1.515	–1.776	27.273	7.994
C(8α)	–5.450	–7.068	–7.414	–6.644
H(8α)	1.635	2.503	8.237	4.125
H(8α)	9.826	10.295	15.582	11.901
H(8α)	25.169	25.435	31.043	27.216
C(8a)	–8.071	–9.664	–14.358	–10.698

Table S5: Hyperfine couplings of TML$^{ox•}$ in MHz.

atom	A_{XX}	A_{YY}	A_{ZZ}	A_{iso}
N(1)	–0.108	0.146	0.608	0.215
C(2)	–0.993	–2.086	–2.454	–1.844
O(2)	1.559	1.693	–8.491	–1.746
N(3)	0.529	–0.547	–0.871	–0.296
H(3)	0.517	–2.244	–2.577	–1.435
C(4)	–14.500	–16.279	–16.714	–15.831
O(4)	4.965	5.421	–23.891	–4.502
C(4a)	–0.275	1.364	87.743	29.611
N(5)	–2.687	–3.299	–8.157	–4.714
C(6)	6.479	7.392	59.385	24.419
C(6α)	1.085	2.163	113.923	39.057
H(6α)	–0.521	–0.892	3.460	0.682
H(6α)	19.964	21.808	24.983	22.252
H(6α)	19.608	21.465	24.521	21.865
C(7)	–22.443	–26.582	–53.291	–34.105
C(7α)	–7.560	–8.087	–8.204	–7.950
H(7α)	–14.145	–35.627	–52.780	–34.184
H(7α)	–14.233	–35.831	–53.653	–34.572
N(8)	1.277	1.418	15.268	5.988
C(8α)	–2.975	–3.618	–3.921	–3.505
H(8α)	2.667	4.201	8.431	5.100
H(8α)	10.378	11.454	14.771	12.201
H(8α)	0.586	1.324	4.002	1.971
C(8a)	–13.510	–15.186	–20.552	–16.416

References

Masuda, T.: Application of chromatography. XXXI. Structure of a green fluorescent substance produced by *Eremothecium ashbyii*, Pharm. Bull., 5, 28-30, 1957.
